# Association among kidney function, frailty, and oral function in patients with chronic kidney disease: a cross-sectional study

**DOI:** 10.1186/s12882-020-02019-w

**Published:** 2020-08-20

**Authors:** Shiho Kosaka, Yuki Ohara, Shotaro Naito, Soichiro Iimori, Hiroshi Kado, Tsuguru Hatta, Masaaki Yanishi, Shinichi Uchida, Makoto Tanaka

**Affiliations:** 1 Critical and Invasive-Palliative Care Nursing, Graduate School of Health Care Sciences, Tokyo Medical and Dental University, 1-5-45 Yushima, Bunkyo-ku, Tokyo, 113-8510 Japan; 2 Tokyo Metropolitan Institute of Gerontology, 35-2 Sakae-cho, Itabashi-ku, Tokyo, 173-0015 Japan; 3 Department of Nephrology, Tokyo Medical and Dental University, 1-5-45 Yushima, Bunkyo-ku, Tokyo, 113-8510 Japan; 4 Omihachiman Community Medical Center, 1379 Tuchida-cho, Omihachiman-city, Shiga 523-0082 Japan; 5 Kansai Medical University, 2-3-1 Shinmachi, Hirakata-city, Osaka, 573-1191 Japan

**Keywords:** Chronic kidney disease, Frailty, Oral function, Kidney function

## Abstract

**Background:**

Chronic kidney disease (CKD) involves many factors that can cause frailty and oral hypofunction. We aimed to investigate the prevalence of frailty and oral hypofunction and to examine the associations among kidney function, frailty, and oral function in adults with CKD in Japan.

**Methods:**

This cross-sectional study was conducted at two institutions. The participants included 109 patients with CKD stages 3–5 who visited outpatient clinics or were admitted for inpatient treatment. Frailty was evaluated using the Japanese version of the Cardiovascular Health Study frailty criteria. Oral function was evaluated by assessing oral motor skills [oral diadochokinesis (ODK) rate], masticatory ability, and the repetitive saliva swallowing test. The estimated glomerular filtration rate (eGFR) was used to indicate kidney function. We examined the associations among kidney function, frailty, and oral function using binomial logistic regression analysis.

**Results:**

In total, 31 participants (28.4%) were classified as being frail. Univariate analysis showed that age, body mass index, eGFR, and haemoglobin level were significantly associated with frailty. ODK and swallowing function were significantly associated with frailty. Multivariate analysis revealed that frailty was significantly associated with eGFR [odds ratio (OR) 0.96, 95% confidence interval (CI) 0.92–1.00, *p* = 0.048] and ODK rate (OR 0.68, CI 0.47–0.98, *p* = 0.038). However, no significant association was found between CKD severity and masticatory or swallowing function.

**Conclusion:**

We found a high prevalence of frailty in patients with CKD and a significant association between frailty and oral motor skills, affecting the swallowing function of patients with nondialysis CKD. The high prevalence of frailty among patients with CKD suggests that routine assessment of frailty is necessary to prevent the development of severe complications. In addition, oral and kidney function should be carefully evaluated, and oral health education and interventions should be performed for patients with CKD.

## Background

Chronic kidney disease (CKD) is a common disease worldwide [1] and is associated with several comorbid diseases, such as hypertension, chronic cardiovascular disease, hyperuricaemia, and metabolic syndrome [2]. Patients with CKD are more than twice as likely to report reduced physical activity than patients without CKD due to systemic abnormalities caused by worsening kidney function. Furthermore, patients with CKD often develop sarcopenia due to muscle weakness caused by protein–energy wasting [3]. In addition, the majority of patients with CKD develop malnutrition before the initiation of dialysis [4–6]; therefore, they have a high risk of developing frailty.

Frailty is a common geriatric syndrome that has been gaining increased attention. In 2001, Fried et al. [7] reported a standardized definition of frailty and created the concept of the frailty phenotype. Frailty is strongly associated with malnutrition, sarcopenia, resting metabolic rate, and decreased energy expenditure [7]. A large, population-based Japanese survey found that 11.3% of elderly participants had symptoms of frailty [8].

The prevalence of frailty among patients with nondialysis CKD ranged from 7.9% [9] to 16% [10], whereas that of frailty among patients with severe CKD was 20.9% [9]. The presence of frailty among patients with CKD has also been associated with a 1.95-fold increase in the risk of all-cause mortality [11]. In addition, frailty decreases quality of life in patients with CKD [12]. Therefore, the prevention of frailty is important for patients with CKD.

Recent studies have indicated that oral function is significantly associated with physical frailty, sarcopenia, disabilities, and mortality in the future [13, 14]. In these studies, affected oral functions associated with frailty included masticatory function and oral motor skills, and these functions were associated with changes in dietary intake. In a study of the oral health status of patients with CKD [15], the prevalence of oral lesions, burning mouth, abnormal taste, halitosis, and xerostomia was significantly higher in patients with kidney disease than in the general population. These symptoms become exacerbated as kidney function deteriorates. In addition, patients with CKD have a high prevalence of periodontal disease [16–18]. Although many factors can cause oral hypofunction in patients with CKD, few studies have focused on the ability to eat. Therefore, we hypothesized that poor oral function might occur in patients with CKD and that these factors could have an impact on their frailty. We aimed to investigate the prevalence of frailty and oral hypofunction relative to CKD severity, as well as to examine the associations among kidney function, frailty, and oral functions in adults with CKD in Japan.

## Methods

### Data collection and study setting

This cross-sectional study was conducted at two institutions (Tokyo Medical and Dental University Medical Hospital and Omihachiman Community Medical Center). Data were collected from patients with CKD who visited outpatient clinics or who were admitted for inpatient treatment between July 2018 and May 2019. This study was approved by the ethics committees of both institutions and was conducted in accordance with the ethical principles of the Declaration of Helsinki. All of the participants provided written informed consent (approval number M2018–021, 30–46).

### Study participants

Patients with CKD who met the inclusion criteria were consecutively enrolled in the survey. The inclusion criteria included the following: age ≥ 20 years old, CKD stages 3–5 [19], current nondialysis status, ability to understand Japanese, ability to walk independently, consent to participate, and agreement of the patient’s physician. The exclusion criteria included anticipated dialysis commencement or kidney transplant within the subsequent 1 month and cognitive impairment.

### Measurements

The frailty phenotype, oral function, and number of teeth were evaluated, and medical records were reviewed. Frailty evaluations and oral function tests were performed by a nurse or dental hygienist who received training on the study protocol and function tests prior to performing the surveys.

#### Frailty phenotype

To evaluate frailty, we used the Japanese version of the Cardiovascular Health Study frailty criteria (J-CHS) [20]. The J-CHS involves data collection based on the following questions or measurements.

Weight loss: “Yes” in response to the question “Have you lost 2 kg or more in the past 6 months?”

Muscle weakness: Grip strength of < 26 kg in men or < 18 kg in women as measured using N-FORCE HG200 (N-FORCE, Wakayama, Japan).

Endurance: “Yes” in response to the question “In the past 2 weeks, have you felt tired without a reason?”

Gait speed: The participants walked a total of 6 m at a usual pace, and the time necessary to traverse 6 m was measured. A walking speed of < 1.0 m/s was defined as low-level mobility.

Physical activity: “No” in response to either of the following questions: “Do you engage in moderate-level physical exercise or sports aimed at health? Do you engage in low-level physical exercise aimed at health?”

Frailty was defined as having 3–5 of the aforementioned domains, as pre-frailty by having 1 or 2, and as robust by having none.

#### Oral function

Oral function was evaluated according to the following three aspects as recommended by the Japanese Society of Gerodontology [21]: oral motor skill, masticatory ability, and repetitive saliva swallowing.

1. Oral motor skill

Oral motor skills were assessed using the oral diadochokinesis (ODK) test. In the ODK test, a participant pronounces sounds as quickly as possible to allow for the evaluation of the lip (/Pa/), tongue tip (/Ta/), and tongue back (/Ka/) movements, collectively referred to as slickness. We measured the ODK rate according to a previously described method [14]. The participants pronounced the three sounds -- Pa, Ta, and Ka -- repeatedly for 5 s each, and the number of times that each sound was pronounced per second was recorded.

2) Masticatory ability test

The masticatory ability test involves the use of a gummy candy containing glucose [22]. Each participant chewed the candy for 20 s without swallowing and used 10 mL of water to rinse the mouth, which were then spat out and collected, including all saliva and the remaining chewed candy. Next, the glucose level in the exudate was measured.

3) Repetitive saliva swallowing

Swallowing function was assessed using the repetitive saliva swallowing test (RSST) [23, 24]. The RSST was performed by recording the number of times that a participant swallowed saliva within a fixed time. An examiner placed a finger on a participant’s pharyngeal protuberance and hyoid bone and asked the participant to repeatedly swallow saliva as many times as possible for 30 s. The number of times that the movement of the pharyngeal protuberance crossed the examiner’s finger pad was recorded.

#### Dental status

Dental status was evaluated as a factor related to oral function. The numbers of present teeth and functional teeth were counted. We defined present teeth as naturally grown teeth and functional teeth as both present and missing teeth replaced by prostheses, such as dentures and dental implants. Teeth with severe decay and those with stumps were excluded from the category of functional teeth because they were not used for mastication.

#### Kidney condition and related factors

Medical records were reviewed to collect information regarding sex, age, kidney function [estimated glomerular filtration rate (eGFR)], laboratory test results (haemoglobin, albumin, triglyceride, and blood glucose levels), comorbidities (diabetes, hypertension, hyperlipidaemia, cardiovascular disease, cerebrovascular disease, hyperuricaemia, and osteoporosis), CKD stage, body height, body weight, body mass index (BMI), and primary kidney disease. eGFR was calculated using the equation from the new Japanese coefficient-modified Modification of Diet in Renal Disease study: eGFR (mL/min/1.73 m^2^) = 194 × (serum creatinine)^− 1.094^ × (age)^− 0.287^ (× 0.739 for women) [25].

### Statistical analysis

Demographic data were verified for normal distributions. Descriptive characteristics are presented as the mean and standard deviation (SD) or the median and interquartile range (IQR) for continuous data and as frequencies and percentages for categorical data. In bivariate analysis, the participants were divided into robust and pre-frail groups (non-frail groups) and compared with the frail group; differences between the two groups were evaluated using the unpaired Student’s *t*-test for parametric variables, the Mann-Whitney *U*-test for non-parametric variables, and the chi-square test for categorical variables. The Jonckheere-Terpstra test was performed to compare CKD stage and oral function. In multivariate analysis, to determine the associations among kidney function, frailty, and oral function, we performed binomial logistic regression analysis. Due to the small number of robust patients and the multiple independent variables used in the study model, we made the dependent variable, i.e., frailty, a binary value (robust and pre-frail vs. frail), and the independent variables used were kidney and oral functions. Variables that could be considered confounding factors based on previous studies [26–28] were entered as adjustment variables among those that became significant in the univariate analysis. To refine multi-collinearity, we calculated the correlation coefficient for each variable, and a strong correlation of ≥0.6 resulted in the removal of one of the variables. All of the analyses were performed using SPSS software, version 25.0 (IBM Corporation, Armonk, NY, USA). For all of the statistical tests, a *p*-value of < 0.05 was considered statistically significant.

## Results

Among the 119 patients who met the inclusion criteria, 109 (91.6%) consented to participate in this study. Table 1 shows the distribution and bivariate analysis findings of the frailty phenotype and related factors. In total, 31 participants (28.4%) were classified as being frail, and the overall age was 70.9 ± 11.3 years old. Regarding the prevalence of comorbidities, 34.5% of the participants had diabetes, 68.2% had hypertension, and 22.0% had cardiovascular disease.

**Table 1:** Participant characteristics according to frailty (*N* = 109)

		Overall	Robust + pre-frail (non-frail) (*n* = 78)	Frail (*n* = 31)	*p-value*
Sex (male), n (%) (a)		72 (66.1)	54 (69.2)	18 (58.1)	0.267
Age, median (IQR) (b)		71 (66–79)	71 (66–78)	78 (69–85)	0.004
BMI, mean ± SD (c)		23.8 ± 3.9	24.3 ± 3.7	22.7 ± 4.0	0.039
CKD stage, n (%) (a)	3a	17 (15.6)	15 (19.2)	2 (11.8)	0.167
	3b	35 (32.1)	27 (34.6)	8 (22.9)	
	4	40 (36.7)	26 (33.3)	14 (35.0)	
	5	17 (15.6)	10 (12.8)	7 (41.2)	
Blood chemistry, mean ± SD, median (IQR)	eGFR (ml/min/1.73 m^2^) (c)	29.8 ± 13.1	31.6 ± 13.1	25.2 ± 12.2	0.02
	Haemoglobin (g/dL) (c)	12.4 ± 1.7	12.6 ± 1.6	11.9 ± 1.8	0.036
	Albumin (g/dL) (b)	3.9 (3.6–4.1)	3.9 (3.6–4.1)	3.9 (3.6–4.1)	0.788
	Triglyceride (mg/dL) (b)	121.0 (98.0–176.0)	119.0 (104.5–176.8)	126.0 (104.5–176.8)	0.644
	Blood glucose (mg/dL) (b)	106.0 (92.0–128.0)	106.0 (91.8–128.8)	110.0 (93.3–128.3)	0.724
Primary kidney disease, n (%) (a)	Glomerulonephritis	24 (22.0)	19 (24.4)	5 (16.1)	0.582
	Diabetic nephropathy	19 (17.4)	13 (16.7)	6 (19.4)	
	Polycystic kidney disease	1 (0.9)	1 (1.3)	0	
	Nephrosclerosis	35 (32.1)	22 (28.2)	13 (41.9)	
	Others	30 (27.5)	23 (29.5)	7 (22.6)	
Comorbidity, n (%) (a)	Diabetes Mellitus	38 (34.5)	24 (30.8)	14 (45.2)	0.155
	Hypertension	75 (68.2)	55 (70.5)	20 (64.5)	0.542
	Hyperlipidaemia	38 (34.5)	29 (37.2)	9 (29.0)	0.421
	Cardiovascular disease	24 (22.0)	14 (17.9)	10 (32.3)	0.104
	Cerebrovascular disease	8 (7.3)	5 (6.4)	3 (9.7)	0.555
	Hyperuricemia	25 (22.7)	18 (23.1)	7 (22.6)	0.956
	Osteoporosis	2 (1.8)	1 (1.3)	1 (3.2)	0.495
Oral function, mean ± SD or median (IQR)	ODK (pa)/sec (b)	6.0 (4.8–6.7)	6.2 (5.6–6.8)	5.3 (4.4–6.5)	0.004
	ODK (ta)/sec (b)	5.8 (4.9–6.4)	6.0 (5.2–6.7)	4.9 (4.0–6.1)	0.001
	ODK (ka)/sec (b)	5.2 (4.0–6.0)	5.6 (4.2–6.2)	4.3 (3.6–5.9)	0.027
	Mastication ability (c)	150.0 ± 61.5	156.9 ± 58.4	132.2 ± 66.6	0.062
	RSST number of times (b)	2.0 (1.0–4.0)	2.5 (1.0–4.3)	1.5 (0–3.0)	0.029
Tooth condition, median (IQR) (b)	Number of teeth	22.0 (9.0–26.0)	23.0 (13.5–27.0)	15.5 (7.8–25.3)	0.11
	Functional teeth	28.0 (25.0–28.0)	28.0 (25.0–28.0)	25.5 (21.0–28.0)	0.015

Data are expressed as mean ± standard deviation or median (inter-quartile range), and compared by χ square test^a^, Mann-Whitney U test^b^, and unpaired t-test^c^. *ODK* Oral diadochokinesis, *RSST *Repetitive saliva swallowing test

The variables that differed significantly between the frail and non-frail groups were age, BMI, eGFR, and haemoglobin level (*p* < 0.05, all). Significant differences were also observed in the ODK rate and RSST findings, with declining values in the frail group. The number of functional teeth differed significantly according to the frailty phenotype (*p* < 0.05). However, no significant differences were observed in sex, albumin level, triglyceride level, blood glucose level, presence of primary kidney disease, or complications between the two groups.

Table 2 shows the descriptive statistics for oral function according to CKD stage. No statistically significant differences were found among the CKD stages for oral function.

**Table 2:** Descriptive statistics for oral function by CKD stage (*N* = 109)

		Overall	Stage 3a	Stage 3b	Stage 4	Stage 5	*p for trend*
Oral function, mean ± SD or median (IQR)	ODK (pa)/sec	6.0 (4.8–6.7)	6.2 (5.8–6.7)	6.0 (4.8–6.8)	6.2 (4.8–6.9)	5.4 (3.5–6.3)	0.113
	ODK (ta)/sec	5.8 (4.9–6.4)	6.0 (5.2–6.5)	5.6 (5.1–6.3)	5.9 (4.9–6.6)	5.6 (4.7–6.2)	0.386
	ODK (ka)/sec	5.2 (4.0–6.0)	5.6 (4.4–6.0)	5.5 (4.0–6.1)	5.2 (3.9–6.2)	5.0 (3.6–6.2)	0.459
	Mastication ability	150.0 ± 61.5	166.0 ± 67.5	153.0 ± 66.0	142.7 ± 55.0	140.1 ± 62.2	0.161
	RSST number of times	2.0 (1.0–4.0)	2.4 ± 1.8	2.7 ± 2.1	2.9 ± 2.5	2.1 ± 1.4	0.746
Tooth condition, median (IQR)	Number of teeth	22.0 (9.0–26.0)	24.5 (15.8–26.0)	21.0 (6.0–26.0)	22.0 (9.8–28.0)	22.0 (10.5–27.0)	0.916
	Functional teeth	28.0 (25.0–28.0)	27.0 (24.0–28.0)	28.0 (25.8–28.0)	28.0 (23.0–28.0)	27.0 (24.0–28.0)	0.717

Jonckheere-Terpstra trend test

*ODK* Oral diadochokinesis, *RSST* Repetitive saliva swallowing test

Table 3 shows the results of the binomial logistic regression analysis, which examined the associations among kidney function, frailty, and oral function after adjusting for age, sex, and BMI. Frailty was significantly associated with eGFR [odds ratio (OR) 0.96, 95% confidence interval (CI) 0.92–1.00, *p* = 0.048] and ODK rate for /ta/ (OR 0.68, 95% CI 0.47–0.98, *p* = 0.038). However, no significant association was found between frailty and either masticatory or swallowing function.

**Table 3:** Associations among frailty, kidney function, and oral function

Parameters	Odds ratio	95% Confidence interval	*p-value*
eGFR	0.96	0.92–1.00	0.048
ODK (ta)/sec	0.68	0.47–0.98	0.038
Mastication ability	1.00	0.99–1.01	0.889
RSST number of times	0.76	0.57–1.01	0.056
Age	1.05	0.99–1.11	0.097
Sex	0.73	0.26–2.02	0.541
BMI	0.92	0.80–1.06	0.263

Binary logistic regression analysis

*ODK* Oral diadochokinesis, *RSST* Repetitive saliva swallowing test

## Discussion

To our knowledge, this study was the first to examine the associations among kidney function, frailty, and oral function, with a specific focus on the functions necessary for swallowing, mastication, and oral motor skills among patients with nondialysis CKD in Japan. The prevalence of frailty in our participants was 28.4%, and kidney function significantly decreased in the frail group compared with the non-frail group. Frailty was significantly associated with decreased oral motor skills and swallowing function according to univariate analysis. However, the significant decline in oral function was not associated with increased severity of CKD. Multivariate analysis showed that decreased kidney function and oral motor skills were independent factors associated with frailty, even after controlling for the effects of age, sex, and BMI.

The prevalence of frailty was much higher in our study than that reported in a previous national survey (prevalence of 11.3% frailty among the elderly living in Japan) [8] and was higher than that reported by another study (prevalence of 20.9% frailty among patients with severe kidney disease) [9]. Previous studies have shown that, as the severity of kidney disease worsens, physical activity decreases [29], and the prevalence of frailty increases with declining kidney function in patients with CKD [30, 31]; our results are consistent with the results of these studies. Based on these results, the high prevalence of frailty among patients with CKD must be better recognized, and patients should be provided with intensive support. Other variables associated with frailty included BMI and haemoglobin level. Previous studies [32, 33] have also found that people with lower BMI have higher risks of frailty, subsequently increasing the risk of death [34]. Low haemoglobin levels (anaemia) have also been associated with a higher risk of developing frailty [35–37], similar to the results of the current study.

Similar to the findings of previous studies [13, 14], frailty was a factor significantly associated with oral hypofunction in our study.

However, when oral function was examined according to CKD stage, no significant differences were found among different CKD stages. Previous studies have primarily focused on periodontal disease and oral dryness in patients with CKD. Decreased saliva volume has been associated with a decline in swallowing function, and periodontal disease can lead to weakened masticatory ability [38–40]. Many patients with CKD present symptoms of periodontal disease [41–43] and hyposalivation [44]; therefore, we investigated whether CKD severity was associated with oral hypofunction. However, no significant association was identified between CKD severity and oral function by univariate analysis. These results could be due to the small number of participants in this study, and the current study did not investigate the extent of xerostomia or periodontal status but only assessed oral function. Therefore, the study population might have included fewer individuals with mechanical insufficiency. Having fewer teeth has also been shown to result in poor masticatory function and malnutrition [45–47]. However, the participants in this study had, on average, > 25 functional teeth. Because no significant differences were observed in the number of teeth according to CKD severity, masticatory ability might have been retained through the maintenance of occlusal status due to proper dental treatment, such as the use of prostheses. In future studies, the comprehensive evaluation of salivary secretion, taste sensitivity, severity of periodontal disease as an infectious disease, and periodontal function should be performed, all of which can affect oral function.

Multivariate analysis showed a significant association among frailty, kidney function, and tongue motor skills. The ODK /ta/ test reflects the movement of the tongue, which carries food to the back of the mouth and is associated with swallowing [48, 49]. In addition, although no significant difference was identified, a tendency towards a declining swallowing function was observed with a decline in kidney function. Therefore, reduced tongue movement and swallowing function, i.e., related to the ability to eat, could occur in patients with CKD. Although these results are limited due to the small study population, the detection of low nutrition intake and other systemic disorders is possible by increasing the number of subjects and performing longitudinal observations of groups with reduced oral function in future studies.

The results of this study suggest that oral function is associated with frailty in patients with CKD and must be carefully monitored. Although patients with CKD have been reported to have lower rates of dental visits than the general population [50], regular dental visits and habitual monitoring of oral function, such as the ability to chew, swallow, and eat, are recommended to help prevent severe frailty. Regular check-ups by multidisciplinary medical staff and the establishment of seamless consultations with a dental professional are required [51]. Healthcare providers must perform comprehensive patient management across disciplines and individual specialties. In addition, the promotion of daily self-care [52, 53] among patients is necessary to educate them so that they can undertake preventive measures.

The present study has several limitations. First, the study design was cross-sectional; therefore, the evidence for causality was limited. Second, although the participants were recruited from two institutions, a small number of participants were enrolled, and they were not necessarily representative of all patients with this disease; therefore, the results might not be generalizable to other populations. In addition, potential factors related to frailty, kidney function, and oral function might not have all been examined and therefore should be further studied with an increased sample size and assessment variables in the future. Third, to understand the nutritional status of patients with CKD, both serum levels and comprehensive nutritional indicators must be evaluated, such as the Subjective Global Assessment score [3, 54] and food intake [55, 56]. Finally, we only examined oral function and the number of teeth; however, the associations of CKD with saliva volume and periodontal disease should also be determined.

Despite these limitations, this study was the first to show the actual state of oral function in patients with CKD, and we believe that long-term studies will clarify the associations between CKD and not only frailty but also mortality and other outcomes. In the future, the comparison between patients with CKD and those without CKD will be necessary to determine precautions specific to CKD and to provide appropriate interventions.

## Conclusions

The findings of this study indicate a high prevalence of frailty among patients with CKD and a significant association between frailty and the oral motor skills that affect swallowing in patients with nondialysis CKD. Recognizing the high prevalence of frailty in patients with CKD and performing routine assessments of frailty will be necessary to prevent the development of severe complications. Moreover, oral function and kidney function should be carefully examined, and early education and interventions regarding oral health should be performed for patients with CKD.

## Data Availability

The datasets used and/or analysed during the current study are available from the corresponding author on reasonable request.

## Abbreviations

BMI: Body mass index
CI: Confidence interval
CKD: Chronic kidney disease
eGFR: Estimated glomerular filtration rate
J-CHS: Japanese version of the Cardiovascular Health Study frailty criteria
ODK: Oral diadochokinesis
OR: Odds ratio
RSST: Repetitive saliva swallowing test
SD: Standard deviation
IQR: Interquartile range
